# A Geriatrics Telehealth Curriculum: A Workshop for Medical Students on “Tele-skills” using Standardized Patients

**DOI:** 10.1093/geroni/igaa057.3352

**Published:** 2020-12-16

**Authors:** Lindsay Wilson, Ross Powell, Megan Foster, Kathleen Vogel, Casey Kelley, Cristine Henage, Ellen Roberts, Jan Busby-Whitehead

**Affiliations:** 1 University of North Carolina, Chapel Hill, North Carolina, United States; 2 ^2.^ University of North Carolina School of Medicine, Chapel Hill, North Carolina, United States

## Abstract

Background: Telemedicine allows for interprofessional care of geriatric patients and allows older adults to access healthcare from their homes. The coronavirus pandemic has prompted a rapid shift to telemedicine. In 2016-2017, only 58% of medical schools in the US offered telemedicine curricula. Thus, a large gap in medical education has emerged. There are specific skills needed to ensure students’ “webside” manner is comparable to their bedside manner.This curriculum was created to train medical students in geriatric-sensitive telemedicine using standardized patients (SP). Methods: A didactic detailing geriatric interviewing preceded the SP encounter. Students were assigned roles for the SP encounter as follows: A) Set agenda, elicit questions, triage problems, perform a history, ensure appropriate lighting and audio B) Perform a geriatric review of systems and reconcile medications C) Present an assessment and plan to the preceptor D) Relay the plan to the SP E) Provide feedback. Students were given pre- and post-surveys to assess their comfort using telemedicine and caring for SP’s >65 years old. Results: Seventeen participants were surveyed (pre-survey=17, post-survey=10). Fifty-nine percent of participants reported no prior experience with telemedicine. Participants reported statistically significant increases in comfort using telemedicine (p=0.022), using telemedicine for patients >65 years old (p<0.001), interviewing patients >65 years old over telemedicine (p=0.007), managing patients over telemedicine (p=0.040), and managing patients >65 years old over telemedicine (p=0.001) after completing the curriculum. Discussion: This virtual curriculum improved medical student comfort with geriatric care and telemedicine and highlights the need for telemedicine curricula in medical schools.

